# Intravitreal Bevacizumab Treatment in Type 2 Idiopathic Macular Telangiectasia

**DOI:** 10.4274/tjo.23921

**Published:** 2016-12-01

**Authors:** Tuğba Aydoğan, Gürkan Erdoğan, Cihan Ünlü, Ahmet Ergin

**Affiliations:** 1 Ümraniye Training and Research Hospital, Ophthalmology Clinic, İstanbul, Turkey

**Keywords:** Type 2 idiopathic macular telangiectasia, intravitreal bevacizumab, Macular edema

## Abstract

**Objectives:**

To evaluate the efficacy of intravitreal bevacizumab treatment in type 2 idiopathic macular telangiectasia (IMT).

**Materials and Methods:**

Six eyes of 5 patients with type 2 IMT who received intravitreal bevacizumab between 2009 and 2014 were included in this study. All the patients had an ophthalmological examination including best corrected visual acuity (BCVA), dilated fundus examination, spectral domain optical coherence tomography (OCT) and fluorescein angiography. Intravitreal bevacizumab injection was planned for patients who had macular edema and/or decreased visual acuity at baseline. Patients were examined 1 week and 1 month after the intravitreal injection. Intravitreal injection was repeated in patients whose visual acuity decreased and/or whose macular edema persisted or increased. Changes in BCVA, central macular thickness (CMT) and central macular volume from baseline at 1 month after the first injection and at final examination were evaluated.

**Results:**

Average age of the patients (4 female and 1 male) was 62±11.8 years. Average follow-up period was 26±11 months. Patients received an average of 2.3 (range 1-4) injections during follow-up. Average Snellen BCVA of the patients was 0.48±0.29. BCVA increased at final examination compared to baseline in all of the patients. The difference between baseline and final visual acuities was significant (p<0.05). The patients’ average CMT was 328±139 µm at baseline and decreased by a mean of 85±153 µm at 1 month after the first injection and 65±142 µm at final examination, but the changes were not significant. CMT decreased at final examination compared to baseline in four patients and increased in both eyes of one patient.

**Conclusion:**

Intravitreal bevacizumab injection is a preferable treatment method in regard to both visual acuity and OCT findings.

## INTRODUCTION

Idiopathic macular telangiectasia (IMT), first described by Gass and Oyakawa,^1^ is a clinical condition of telangiectasia and aneurysmal dilatations of the juxtafoveal retinal capillaries. IMT type 2 affects both genders equally and is more common in the fifth and sixth decades. Telangiectatic changes are the most common changes seen in the fundus. Although patients may initially present with unilateral involvement, long-term follow-up usually reveals changes in the fellow eye as well.^2^ Yannuzzi et al.^3^ separated IMT into nonproliferative and proliferative subgroups.

Clinical findings are highly variable; mild cases may manifest as loss of retinal transparency in the perifoveal temporal region, while more severe cases exhibit prominent telangiectatic vessels on fundoscopy, right-angle venules, intraretinal crystalline deposits, retinal pigment epithelium cell migration, and ultimately transformation to the proliferative type.^2,3^ On fluorescein angiography (FA), slight intraretinal staining is observed in the early disease stages, whereas patients with substantial telangiectatic changes exhibit filling of the superficial telangiectatic capillaries and leakage from the deep capillaries.^3^ Increased foveal thickness and intraretinal cystoid changes may be observed on spectral domain optical coherence tomography (SD-OCT).^3,4,5,6^ Other possible findings are outer retinal atrophy and disruption of the inner segment/outer segment junction.^5,6^

Various treatments such as focal/grid argon laser therapy,^7^ transpupillary thermotherapy,^8^ photodynamic therapy,^9^ subretinal membrane surgical excision,^10^ and intravitreal triamcinolone^11,12^ have been tried in type 2 IMT patients. In recent years, intravitreal anti-vascular endothelial growth factor (VEGF) injection has been administered to proliferative and nonproliferative patient groups in a variety of studies.^12,13,14,15,16,17,18,19,20,21,22,23,24^ Although the results of these studies differ, some patients reportedly benefited from intravitreal anti-VEGF injections.

In the present study we aimed to examine the functional and morphologic effects of intravitreal bevacizumab injection in type 2 IMT patients.

## MATERIALS AND METHODS

The study included 6 eyes of 5 patients treated with intravitreal bevacizumab therapy and followed in our clinic for type 2 IMT between 2009 and 2014. Approval was granted by the local ethics committee and informed consent forms were obtained from all patients.

All patients underwent a full ophthalmologic examination including best corrected visual acuity (BCVA) measurement and dilated fundus examination, SD-OCT (RTVue; Optovue Inc, CA, USA) and FA (Visucam; Zeiss, Meditec, Germany). Visual acuity was measured using Snellen chart and converted to logMAR (logarithm of the minimum angle of resolution) for statistical analysis. OCT measurements were done using a MM5 (5x5 mm2 grid) protocol. Intravitreal bevacizumab injection was indicated in patients with macular edema and/or reduced visual acuity at presentation.

Intravitreal injections were performed in sterile operating room conditions. Intravitreal bevacizumab (1.25 mg) (Avastin, Roche, Germany) injections were done using a 27-gauge needle applied 3.5 mm from the temporal limbus in phakic patients and 3 mm in pseudophakic patients. Follow-up examinations were conducted at 1 week and 1 month after intravitreal injection. FA was repeated an average of once every 3 months. Intravitreal bevacizumab injections were repeated in patients whose BCVA decreased and/or whose macular edema persisted or worsened.

BCVA, central macular thickness (CMT) and central macular volume (CMV) were compared at baseline, at 1 month after the first injection and at final examination.

### Statistical Analysis

Number Cruncher Statistical System 2007&PASS (Power Analysis and Sample Size) 2008 Statistical Software (Utah, USA) was used for all statistical analyses. Study data were evaluated using descriptive statistical methods (mean, standard deviation, median, minimum and maximum) and the paired-samples t-test was used to compare quantitative data. Level of significance was p<0.05.

## RESULTS

Mean age of the patients (4 female and 1 male) was 62±11.8 years. Lesions were nonproliferative in all cases. Mean follow-up time was 26±11 months, during which patients received an average of 2.3 (range 1-4) injections. Patients’ BCVA, CMT and CMV values at baseline, 1 month after the first injection and at final examination are shown in Table 1.

**Figure 1:**
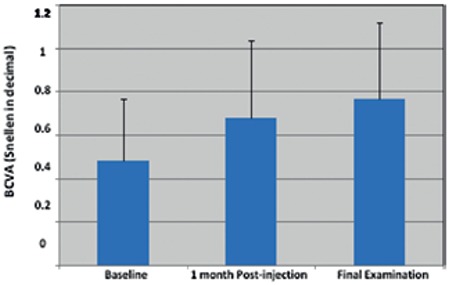
Changes in patients’ mean best corrected visual acuity BCVA: Best corrected visual acuity

Mean Snellen BCVA (expressed as decimal) was 0.48±0.29 at baseline, 0.68±0.36 at 1 month after first injection and 0.77±0.35 at final examination (Figure 1). There was no significant difference in BCVA at 1 month after first injection compared to baseline, but the increase in BCVA between baseline and final examination was significant (p<0.05). All patients’ showed improved BCVA at final examination compared to baseline.

**Figure 2:**
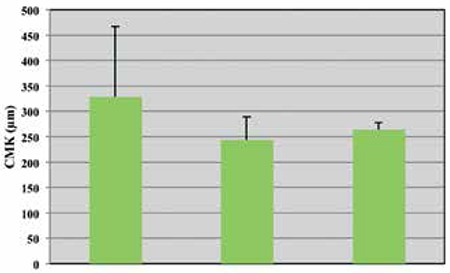
Changes in patients’ mean central macular thickness CMK: Central macular thickness

Mean CMT value was 328±139 µm at baseline, and decreased by a mean of 85±153 µm at 1 month after first injection and by a mean of 65±142 µm at final examination (Figure 2). However, the reductions in CMT were not statistically significant. CMT decreased in 4 patients at final examination compared to baseline, but increased in both eyes of the other patient. No significant changes in mean CMV were observed during follow-up.

Following intravitreal injection, patient 1’s Snellen BCVA improved to 20/20 and OCT revealed that the extrafoveal intraretinal cysts had resolved. The juxtafoveal telangiectatic changes observed on FA diminished but did not completely resolve. There were no changes in the patient’s BCVA during follow-up, so no further injections were administered.

After the first intravitreal injection, patient 2’s Snellen BCVA improved from 20/100 to 20/25, the intraretinal cysts seen on OCT shrank, and a reduction in the juxtafoveal telangiectatic structures was observed on FA. The patient’s BCVA declined during follow-up and another intravitreal injection was administered. Following the second intravitreal injection, BCVA remained stable at 20/25; therefore, no further injections were performed.

**Figure 3:**
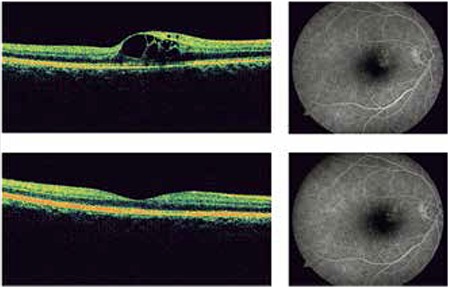
Optical coherence tomography and fluorescein angiography images obtained from patient #3 at presentation and at 1 month after intravitreal bevacizumab injection

Following the first intravitreal injection, patient 3’s Snellen BCVA improved to 20/20, the extrafoveal intraretinal cysts detected by OCT completely resolved, and foveal contours returned to normal. FA showed that the amount of leakage was reduced (Figure 3). Two additional injections were administered during follow-up due to decreased BCVA and increased CMT. After the final injection, BCVA remained stable at 20/20 and the foveal contours returned to normal.

Following the first intravitreal injection, patient 4’s Snellen BCVA remained at 20/400. The intraretinal cysts were smaller on OCT, CMT was substantially decreased and the degree of leakage seen on FA was reduced. Repeated injections were done because the patient’s CMT increased again during follow-up. There were no significant changes in BCVA during follow-up. This was attributed to the development of retinal atrophy due to prolonged macular edema.

In patient 5, BCVA improved in both eyes after intravitreal injection. OCT at final examination revealed slightly increased CMT in both eyes, but the intraretinal cysts were smaller in size. Reduced leakage was observed in both eyes on FA. Additional injections were applied to the patient’s left eye due to reduced visual acuity. Visual acuity in the right eye remained stable after a single injection.

## DISCUSSION

The pathogenesis of type 2 IMT and the role of VEGF molecules in that pathogenetic process continues to be a controversial topic. Yannuzzi et al.^3^ posited that endothelial cell degeneration may be the triggering factor of vasogenic mechanisms in the absence of pronounced ischemia or inflammation. Other investigators have claimed that, considering the function of Müller cells in supporting the retina, dysfunction in these cells may initiate and accelerate endothelial cell degeneration.^25,26^ In their histopathologic study, Green et al.^27^ proposed that endothelial degeneration and capillary structural disruption lead to retinal hypoxia, which may increase VEGF release and angiogenic activity.

Most studies of intravitreal injection of anti-VEGF agents in type 2 IMT have demonstrated that leakage on FA is generally reduced after injection.^12,15,16,17,18,19,20,21,22^ In some of these studies, however, the leakage on FA was reported to return to baseline levels during periods without injections.^15,17,20,22^ Similarly, though decreases in macular thickness measured by OCT may be detected initially,^12,16,17,18,19,20,21,22,24^ studies with long-term follow-up after the final injection reported that OCT findings also returned to baseline.^17,18,20,22^ Besides these studies, there are others in which no substantial changes in OCT findings were observed.^13,14,15^ Results concerning visual acuity vary. Some studies show improvements in visual acuity,^12,18,19,20,22^ whereas others report no change or even decline over time.^13,14,15,16,20,22,24^ Response to treatment varies in terms of disease duration and severity, and degree of neuroretinal degeneration.

In the present study, the finding which most strongly supports intravitreal anti-VEGF therapy is the significant improvement in visual acuity at final examination. Although the patients showed some improvement in OCT findings, the changes were nonsignificant. This may be due to the small number of patients. The better results achieved by some patients may be attributable to factors such as individual differences in treatment response, disease duration, and previous therapies. Despite variation in extent of treatment response, our study demonstrates that intravitreal anti-VEGF is a preferable treatment for type 2 IMT in terms of both visual acuity and OCT findings.

To date, no treatment protocol has been developed for type 2 IMT. Several treatment modalities are being tested. Studies of intravitreal injection of anti-VEGF agents have yielded conflicting data regarding treatment outcomes. Future studies including larger patient groups may provide results which more clearly demonstrate treatment response.

## CONCLUSION

In the present study and others in the literature, there are patients who have clearly benefited from intravitreal anti-VEGF therapy. Therefore, patients should be evaluated individually during the course of disease management.

### Ethics

Ethics Committee Approval: It was taken. Informed Consent: Obtained.

Peer-review: Externally peer-reviewed.

## Figures and Tables

**Table 1:**
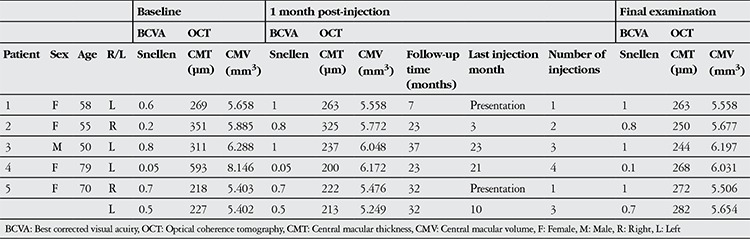
Clinical data of type 2 idiopathic macular telangiectasia patients treated with intravitreal bevacizumab
